# Protective Effects of Miswak (*Salvadora persica*) against Experimentally Induced Gastric Ulcers in Rats

**DOI:** 10.1155/2018/6703296

**Published:** 2018-07-09

**Authors:** Mohamed A. Lebda, Ali H. El-Far, Ahmed E. Noreldin, Yaser H. A. Elewa, Soad K. Al Jaouni, Shaker A. Mousa

**Affiliations:** ^1^Biochemistry Department, Faculty of Veterinary Medicine, Alexandria University, Alexandria 22758, Egypt; ^2^Biochemistry Department, Faculty of Veterinary Medicine, Damanhour University, Damanhour 22511, Egypt; ^3^Histology and Cytology Department, Faculty of Veterinary Medicine, Damanhour University, Damanhour 22511, Egypt; ^4^Histology and Cytology Department, Faculty of Veterinary Medicine, Zagazig University, Zagazig, Egypt; ^5^Laboratory of Anatomy, Department of Biomedical Sciences, Graduate School of Veterinary, Hokkaido University, Sapporo, Japan; ^6^Department of Pediatric Hematology/Oncology, King Abdulaziz University Hospital and Scientific Chair of Yousef Abdul Latif Jameel of Prophetic Medicine Application, Faculty of Medicine, King Abdulaziz University, Jeddah 21589, Saudi Arabia; ^7^Pharmaceutical Research Institute, Albany College of Pharmacy and Health Sciences, Rensselaer, NY 12144, USA

## Abstract

Gastric ulcers are among the most broadly perceived illnesses affecting individuals. Alcohol consumption is the main cause of gastric ulceration. This study assessed the protective effects of *Salvadora persica* (SP) extract against ethanol-induced gastric ulcer and elucidated the conceivable underlying mechanisms involved. For this purpose, 40 rats were allotted into 4 equal groups (control, ethanol- (EtOH-) treated, and SP-treated “SP200 and SP400” groups). The control and EtOH-treated groups were given phosphate buffer saline (PBS), and both the SP200 and SP400 groups were given SP extract dissolved in PBS at doses of 200 and 400 mg/kg b.w., respectively. All treatments were given orally for 7 constitutive days. On the 8th day, all rats were fasted for 24 h followed by oral gavage of PBS in the control group and chilled absolute ethanol solution (5 ml/kg b.w.) in the EtOH- and SP-treated groups to induce gastric lesions. One hour later, the rats were sacrificed and the stomachs were harvested. Gross and microscopic examinations of the EtOH-treated group showed severe gastric hemorrhagic necrosis, submucosal edema, destruction of epithelial cells, and reduced glycoprotein content at the mucus surface. These pathological lesions were defeated by SP extract treatment. Administration of SP extract modulated the oxidative stress and augmented the antioxidant defenses. The elevated ethanol-expressed tumor necrosis factor-*α* (TNF-*α*) and interleukin-1*β* (IL-1*β*) genes, as well as bcl-2-like protein 4 (Bax) and inducible nitric oxide synthase (iNOS), were diminished in the SP-treated group. Curiously, SP extract upregulated endothelial nitric oxide synthase (eNOS) and transforming growth factor-*β*1 (TGF-*β*1) gene expression comparable to that of the EtOH-treated rats. Aggregately, SP exerted antiulcer activities in ethanol-induced gastric ulcer rat models via modulation of oxidant/antioxidant status, mitigation of proinflammatory cytokines, and apoptosis, as well as remodeling of both NOS isoforms.

## 1. Introduction

Gastric ulceration mainly occurs as a result of disharmony between 5 inverse factors at the gastric mucosa [1]. Inducing factors include alcohol, nonsteroidal anti-inflammatory drugs, smoking, stress, and *Helicobacter pylori* [2]. This is in contrast to the gastroprotective factors that are attributed to adequate secretion of mucus and prostaglandins, maintenance of anti-inflammatory and antioxidative agents, and normal mucosal blood flow [3].

Ethanol is one of the forceful factors that prompt gastric ulcer and is used as a model for assessment of the gastro-defensive effects of various drugs and natural products [4]. The oxidative stress that leads to the production of reactive oxygen species (ROS) along with the decline in antioxidative enzymes at the gastric mucosa induced by ethanol ingestion is implicated in the pathogenesis of ethanol-induced gastric ulceration [5]. Alcohol consumption induces gastric mucosal damage and apoptosis through tumor necrosis factor-*α* (TNF-*α*) signaling and ROS formation [6]. TNF-*α*, an initiating proinflammatory cytokine, has a critical role in the pathogenesis of gastric ulcer via inflammation and injury inducement [7]. Alcohol-induced gastric damage has been mediated through hypersecretion of gastric acid [8], proinflammatory cytokines and ROS generation [9], apoptosis induction, and depletion of nitric oxide (NO) and prostaglandin E2 [10]. NO, a vasodilator that is synthesized from the amino acid arginine by two NO synthases, has a dual function at the gastric mucosal level. One of them is the endothelial nitric oxide synthase (eNOS) that produces NO to assist gastric ulcer healing mainly through stimulation of blood vessels' formation, increasing blood flow, and anti-inflammatory action [11], while NO generated from inducible nitric oxide synthase (iNOS) functions in gastric ulcer induction via the formation of ROS and toxic effects on cells [12].

Alleviation of gastric aggressive mediators and progression of gastric preservative factors are considered as therapeutic tools for the healing of gastric ulcer [13]. The mechanism of the healing process encompasses the restoration of the gastro-defensive factors' balance, generation of gastric mucosal cells and blood vessels, matrix reconstruction, antioxidation, and anti-inflammation [14].

Natural products have attracted scientific attention as prophylactic alternatives for gastric ulcer [15]. *Salvadora persica* L. (SP), also known as miswak, a Salvadoraceae family member, has been used mainly as natural toothbrushes [16]. SP-lyophilized decoction has a protective action on gastric ulcer induced by acetylsalicylic acid in rats [17]. Soliman et al. [18] reported that SP root extract attenuated oxidative stress, restored antioxidant enzymes, and reduced glutathione (GSH) level in rats exposed to lead acetate. Also, Nomani et al. [19] showed that the anti-inflammatory activity of SP extract is mediated via the downregulation of TNF-*α* mRNA expression in inflammatory bowel disease-induced rats. Further, the most potent inflammatory factors IL-1*β*, TNF-*α*, and TGF-*β*1 were decreased in rat serum subjected to carrageenan-induced paw edema pretreated with SP extract [20].

In view of previously published data concerning the mechanistic factors of gastric ulcer healing, the current study was designed to evaluate the effects of SP aqueous extract on proinflammatory cytokines, nitric oxide synthases, apoptotic pathways, and oxidative/antioxidative pathways involved in ethanol-induced gastric ulcer in rats.

## 2. Materials and Methods

### 2.1. Ethics Statement

The experiments were done in compliance with the rules set by the Ethics Committee at the Faculty of Veterinary Medicine, Alexandria University, Egypt. All efforts were made to minimize the suffering of rats during experimentation and sampling.

### 2.2. Chemicals and Reagents

Absolute ethanol (EtOH) solution was purchased from Sigma-Aldrich Co. (St. Louis, MO, USA). Polyclonal rabbit anti-mouse iNOS antibody (1 : 100; Cat: ab15323), monoclonal rabbit anti-human Bax antibody (1 : 300; Cat: ab32503), polyclonal rabbit anti-mouse IL-1*β* antibody (1 : 250; Cat: ab9722), and polyclonal rabbit anti-human CD3 antibody (1 : 100; Cat: ab5690) were purchased from Abcam Co., Cambridge, UK.

Kits for malondialdehyde (MDA), GSH, total superoxide dismutase (T.SOD), catalase (CAT), glutathione peroxidase (GPx), and glutathione S-transferase (GST) were obtained from Biodiagnostic Co. (Dokki, Giza, Egypt). Total RNA extraction and SYBR Green Master Mix kits were purchased from Qiagen Co., Germany. cDNA kit was obtained from Promega Co., Madison, WI, USA.

### 2.3. Plant Extraction

SP roots were purchased from the local market in Alexandria, Egypt, and authenticated at the Botany Department, Agriculture Faculty, Alexandria University. One kg of SP roots was cut into small pieces, air dried, and ground into fine powder and then extracted in distilled water for 48 h at 25°C. After centrifugation at 1435 ×g for 15 min, the resulting supernatants were filtered through Whatman number 1 filter paper and the filtrates were concentrated using a rotary evaporator at 40°C [21]. The obtained fine extract powder was subdivided into small portions in brown bottles and freshly prepared before supplementation to rats.

### 2.4. Gas Chromatography-Mass Spectrometry (GC-MS) Analysis of SP Phytochemicals in Extract

SP extract was dissolved in N,O-Bis(trimethylsilyl)trifluoroacetamide and injected into a Trace GC Ultra-ISQ mass spectrometer with a direct capillary column TG-5MS (30 m × 0.25 mm × 0.25 *μ*m). The GC was equipped with splitless mode/30 s with helium as a carrier gas. The temperature of the GC oven was maintained at 45°C for 30 s. The oven temperature was gradually raised to 235°C at a rate of 8°C/min and maintained at 235°C for 7.07 min. The MS ion source temperature was 150°C and mass spectra were obtained at 70 eV [22]. Separated compounds were identified by comparing their mass spectra to the Wiley Registry 8e.

### 2.5. Animal Study Design and Induction of Gastric Ulcer

Forty male adult rats weighing between 240 and 250 g were purchased from the Animal Breeding Unit, Medical Research Institute, Alexandria University. Animals were housed in clean metal cages under optimum conditions proportionate to the Institutional Guideline for Care and Use of Laboratory Animals: temperature: 21 ± 2°C, humidity: 56 ± 5%, 12 h light/dark cycle, and free access to water and to diet as listed in Table 1.

Rats were allocated into the control group (*n* = 20), SP200 (*n* = 10) group that received SP extract 200 mg/kg b.w., and SP400 (*n* = 10) group that received SP extract 400 mg/kg b.w. [23, 24]. SP extract was dissolved in phosphate buffer saline (PBS) and given orally to the SP200 and SP400 groups for 7 days while the control group received only PBS. On the 8th day, all rats were fasted for 24 h followed by administration of ethanol by gastric tube to induce gastric lesions to 10 rats of the control group and all rats in the SP200 and SP400 groups; rats were gavaged with chilled absolute ethanol solution 5 ml/kg b.w. according to the method described by Park et al. [25]. The remaining 10 rats of the control group were gavaged with PBS and kept as negative control. One hour following the induction of gastric lesions, the rats were sacrificed under anesthesia with intravenous injection of sodium pentobarbital (30 mg/kg).

### 2.6. Macroscopic Examination of Gastric Mucosa

Stomachs of anesthetized rats from each group were opened along the greater curvature and rinsed with normal saline (NaCl 0.9%) followed by gross examination for assessment of any abnormal lesions and then photographed. The length of each lesion in mm was measured according to Bozkurt et al. [26], and the gastric ulcer index (UI) was calculated according to the method described by Das and Banerjee [27].

### 2.7. Histological Screening of Gastric Mucosa

Stomachs were flushed with PBS pH 7.4 and fixed in 4% paraformaldehyde in 0.1 M phosphate buffer. Fixed specimens were processed using the conventional paraffin embedding technique including dehydration through ascending grades of ethanol and clearing in 3 changes of xylene and melted paraffin and ended by embedding in paraffin wax at 65°C. Paraffin blocks were sectioned into 4 *μ*m thickness sections. These sections were stained with hematoxylin and eosin (H&E) stain according to the method described by Bancroft and Layton [28] and Periodic acid–Schiff (PAS) stain according to Pearse [29]. The section images were taken with a digital camera (Leica EC3, Leica, Germany) connected to a microscope (Leica DM500).

### 2.8. Determination of Gastric Mucosal Malondialdehyde and Antioxidant Parameters

Gastric tissues were homogenized with PBS to prepare 10% (*w*/*v*) homogenate and divided into 3 aliquots; one was used for the estimation of the MDA level. The second aliquot was deproteinized by adding 10% trichloroacetic acid and centrifuged, and the supernatant was used for the determination of reduced glutathione level, while the third aliquot was centrifuged and used to determine the antioxidant enzyme activities in the supernatant. All procedures were performed using commercial kits (Biodiagnostic Co.) according to the manufacturer's instructions.

### 2.9. Relative Expression of Proinflammatory Cytokine and Endothelial Nitric Oxide Synthase

Total RNA was isolated from 100 mg gastric tissue samples in all groups using an RNA extraction kit (Qiagen Inc., Germantown, MD, USA). qRT-PCR was performed in a real-time PCR machine using one-step SYBR Green RT-PCR Master Mix (Qiagen Inc.) and ready-made primers of TNF-*α*, IL-1*β*, TGF-*β*1, eNOS, and *β*-actin as housekeeping reference genes (Table 2). Thermal cycling conditions were retention time step at 50°C/10 min for cDNA synthesis followed by inactivation step at 95°C/15 min. For gene amplification, conditions were 45 cycles of 95°C/15 s, 58–60°C/30 s, and 60°C/1 min, followed by 60°C/10 min. Analysis of relative gene expression was estimated using the 2^−∆∆Ct^ method [30].

### 2.10. Immunohistochemical Examination of Bax, iNOS, IL-1*β*, and CD3 Proteins

Briefly, 4 *μ*m thick paraffin sections were prepared and deparaffinized using xylene, rehydrated in graded alcohols, and finally washed with distilled water. Antigen retrieval was done in the case of anti-iNOS, anti-Bax, and anti-CD3 by heating in 10 mM citrate buffer (pH 6.0) for 20 min at 95°C with no antigen retrieval for anti-IL-1*β*, followed by washing with distilled water. Deactivation of endogenous peroxidase was carried out using 3% H_2_O_2_ in absolute methanol for 30 min at 4°C. After washing with PBS, the nonspecific reaction was blocked with 10% normal blocking serum for 60 min at room temperature. The sections were incubated at 4°C overnight with the specific primary antibody: monoclonal rabbit anti-human Bax antibody (Abcam, Cat: ab32503); polyclonal rabbit anti-mouse iNOS antibody (Abcam, Cat: ab15323, Cambridge, UK); polyclonal rabbit anti-mouse IL-1*β* antibody (Abcam, Cat: ab9722); and polyclonal rabbit anti-human CD3 antibody (Abcam, Cat: ab5690) diluted in 1.5% BSA/PBS (pH 7.2) at 1 : 100; 1 : 300; 1 : 250; and 1 : 100, respectively. For negative control sections, PBS was used instead of the primary antibody. After washing with PBS, the sections were incubated with biotin-conjugated goat anti-rabbit IgG antiserum (Histofine kit, Nichirei Corp.) for 60 min and then washed with PBS, followed by incubation with streptavidin-peroxidase conjugate (Histofine kit, Nichirei Corp.) for 30 min. The streptavidin-biotin complex was visualized with 3,3′-diaminobenzidine tetrahydrochloride (DAB) H_2_O_2_ solution, pH 7.0, for 3 min. Then sections were washed in distilled water and Mayer's hematoxylin was used as a counterstain. The section images were taken with a digital camera (Leica EC3, Leica, Germany) connected to a microscope (Leica DM500).

### 2.11. Statistical Analysis

Data were analyzed with one-way ANOVA followed by Tukey's post hoc test multiple comparisons using GraphPad Prism 5 (GraphPad Software, San Diego, CA, USA). The data of oxidative and antioxidant status were analyzed with one-way ANOVA followed by Duncan's post hoc test multiple comparisons using the SPSS programming tool (IBM SPSS. 201, Coppell, TX, USA).

## 3. Results

### 3.1. GC-MS Analysis of *S. persica* Extract

Phytochemical ingredients of SP extract detected with GC-MS analysis are listed in Table 3 and Figure S1. The SP extract contained many ingredients with antioxidant potentials such as lycopene (16.56%), *α*-linolenic acid (1.77%), oleic acid (1.97%), lycoxanthin (1.61%), and retinoic acid (1.31%).

### 3.2. Macroscopic Examination of Gastric Mucosa

**Figure 1:**
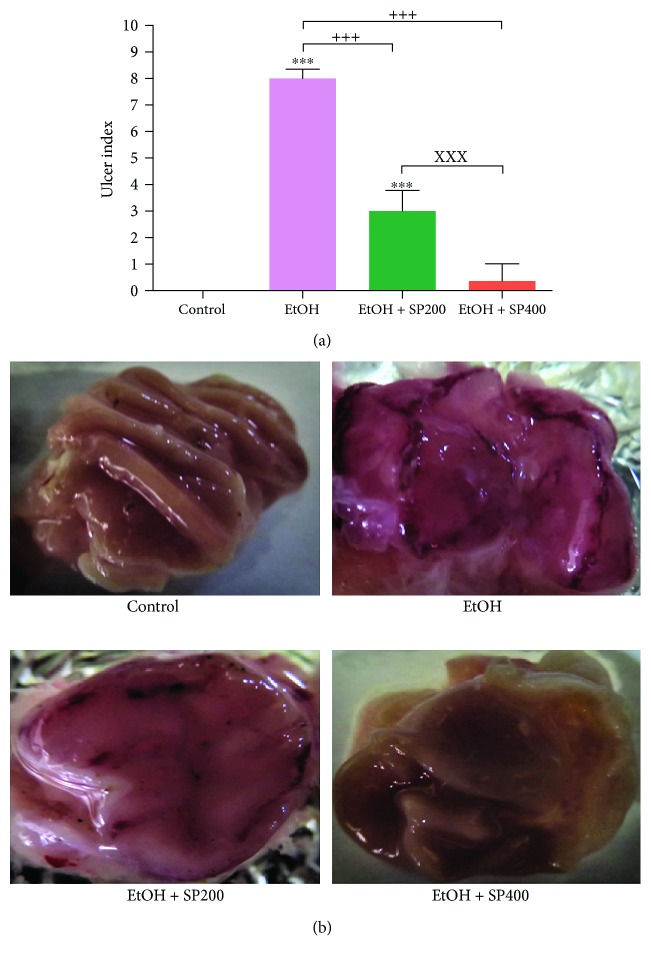
Gastric ulcer index (a) and macroscopic finding of gastric mucosal lesions (b) in rats exposed to ethanol-induced gastric ulcer and treated with *S. persica* extract. EtOH: ethanol-induced gastric ulcer; EtOH + SP200: ethanol-gastric ulcer treated with *S. persica* extract 200 mg/kg b.w.; EtOH + SP400: ethanol-gastric ulcer treated with *S. persica* extract 400 mg/kg b.w. Data are expressed as mean ± SE (*n* = 5), and the statistical analysis was done with one-way ANOVA, followed by Tukey's post hoc test multiple comparisons. ^∗∗∗^ *P* < 0.001 versus control, ^+++^ *P* < 0.001 versus EtOH, and ^xxx^ *P* < 0.001 versus EtOH + SP400.

Normal appearance of gastric mucosal epithelium and folding in the control group is shown in Figure 1. Rats exposed to EtOH revealed severe gastric mucosal congestion and hemorrhage, loose mucosal folds, and thinning and ballooning of the gastric wall with serious ulcers. However, treatment with SP extract significantly reduced the ethanol-induced gastric lesions, moderated congestion and petechial hemorrhage, improved the gastric mucosal folding, and lowered the ulcer index at an SP extract dose of 200 mg/kg, while the high dose of 400 mg/kg caused more alleviation in these lesions as compared to normal ones.

### 3.3. Histological Findings

**Figure 2:**
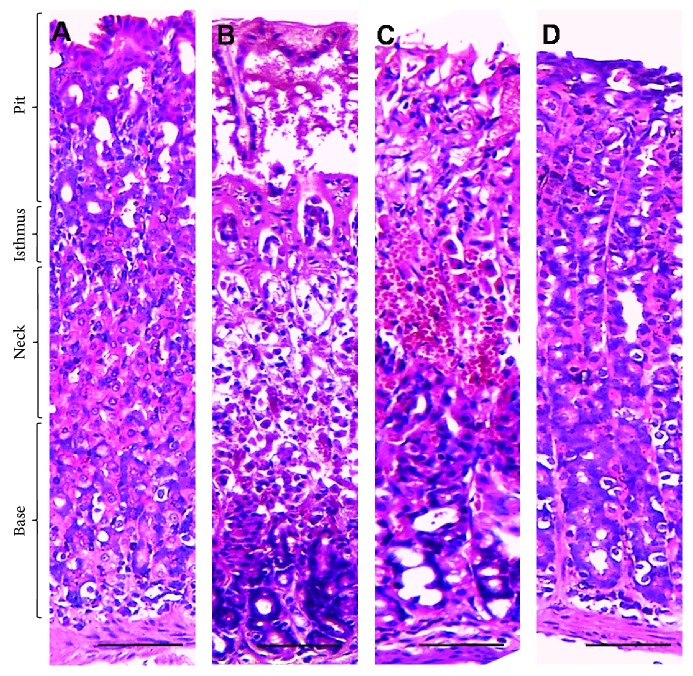
Light microscopic images of gastric mucosa. (a) The control group showed normal histologic appearance of all parts of the gastric wall. (b) The EtOH-induced gastric ulcer group revealed severe degeneration, necrosis, and hemorrhage of gastric base, neck, and isthmus with sloughing of gastric pits. (c) The EtOH-induced gastric ulcer and treated with *S. persica* at a dose of 200 mg/kg b.w. group showed moderated degeneration and hemorrhage in the gastric neck, isthmus, and pits with slight vacuolation in the gastric neck. (d) The EtOH-induced gastric ulcer and treated with *S. persica* at a dose of 400 mg/kg b.w. group showed normal histologic appearance of all gastric portions with only slight vacuolation. Scale bar = 50 *µ*m.

Light microscopic examination of the gastric mucosa stained with H&E showed a normal structure of all gastric portions in the control group (Figure 2). Contrary to the control, ethanol produced gastric damage as manifested by intense degeneration, necrosis, and hemorrhages in almost all parts of the gastric wall, severe submucosal edema, and sloughing of gastric pits. Interestingly, treatment with SP extract significantly reduced the degeneration and hemorrhage induced by ethanol, indicating protective action that was evident with the high dose of 400 mg/kg b.w.

**Figure 3:**
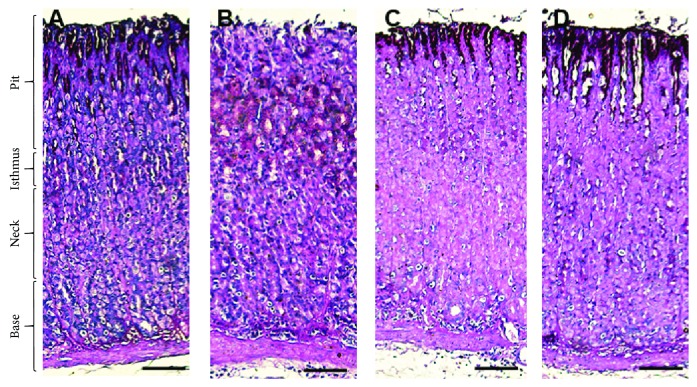
Light micrograph of histochemical staining of periodic acid–Schiff (PAS) in rats. (a) Control rats revealed intense PAS reaction at the surface mucous epithelium in the pit region. (b) No PAS reaction at the surface mucous epithelium in the pit region and moderate PAS reaction at the mucous cells in the pit and isthmus region of EtOH-induced gastric injury. (c) Lower distribution of PAS reacting cells in the SP200-protected group than those observed in the control group concentrated mainly in the pit region. (d) Similar distribution of PAS reacting cells in the SP400-protected group to the control group concentrated in the pit region. Scale bar = 50 *µ*m.

An augmented level of PAS staining was observed at the surface of the mucosal epithelium in the pit region in the control group (Figure 3), indicating high glycoprotein contents. However, negative PAS staining of the surface mucosal epithelium showed low reactivity at the mucosa cells in the pit and isthmus regions among those in the alcohol-treated group. Notably, there was moderate distribution of PAS-reacting cells mainly at the pit region in SP200-treated rats, and an intense reaction was observed in SP400-treated rats resembling those in the control rats.

### 3.4. Oxidative Damage and Antioxidative Biomarkers

The levels of antioxidant GSH (14.2 ± 1.4 *μ*mol/g tissue) and CAT (9.8 ± 1.1 U/g tissue) were significantly reduced in the EtOH group relative to control. Notably, SP extract intake in a dose-dependent manner significantly attenuated the gastric MDA level and motivated the enzymatic and nonenzymatic antioxidant levels *P* < 0.05 when compared to EtOH-treated rats, suggesting antioxidative characteristics of the SP extract (Table 4).

### 3.5. Gastric IL-1*β*, TNF-*α*, TGF-*β*1, and eNOS Relative Expression

**Figure 4:**
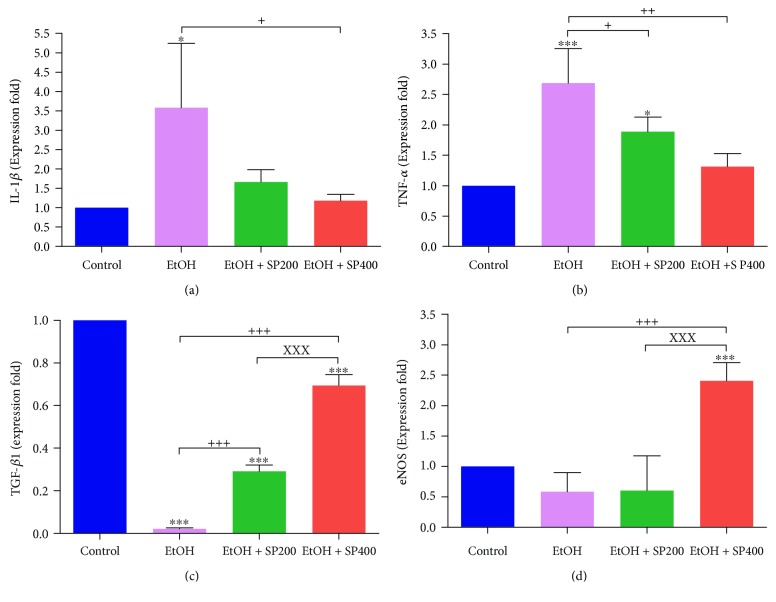
Reverse transcription polymerase chain reaction (RT-PCR) validation of (a) IL-1*β*, (b) TNF-*α*, (c) TGF-*β*1, and (d) eNOS. IL-1*β*: interleukin-1 beta; TNF-*α*: tumor necrosis factor-alpha: TGF-*β*1: transforming growth factor beta; eNOS: endothelial nitric oxide synthase; CON: control; EtOH: ethanol-treated group; EtOH + SP200: ethanol-treated and treated with *S. persica* at a dose of 200 mg/kg b.w.; EtOH + SP400: ethanol-treated and treated with *S. persica* at a dose of 400 mg/kg b.w. ^∗^ *P* < 0.05 and ^∗∗∗^ *P* < 0.001 versus control. ^+^ *P* < 0.05, ^++^ *P* < 0.01, and ^+++^ *P* < 0.001 versus EtOH. ^xxx^ *P* < 0.001 versus EtOH + SP400. Statistical analysis was done with one-way ANOVA, followed by Tukey's post hoc test multiple comparisons. Error bars represent SE. Samples (*n* = 5).

Ethanol intake significantly upregulated the proinflammatory cytokines' expression: TNF-*α* (2.7-fold) (*P* < 0.001) and IL-1*β* (3.5-fold) (*P* < 0.05). It also reduced the expression of TGF-*β*1 (0.02-fold) (*P* < 0.001) and insignificantly downregulated eNOS (0.6-fold), elucidating the inflammatory condition as compared to control (Figure 4). Pretreatment with SP extract significantly downregulated both proinflammatory cytokines in a dose-dependent manner comparable to the EtOH-treated group while causing an elevation in the relative expression of TGF-*β*1 and eNOS *P* < 0.001, which is more pronounced at the high dose of SP extract in comparison to the control group.

### 3.6. Immunohistochemical Detection of Bax, iNOS, IL-1*β*, and CD3 Proteins

**Figure 5:**
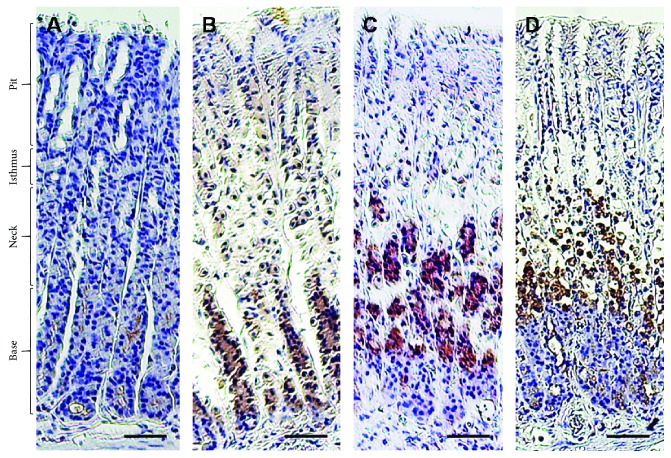
Immunohistochemical reactivity of Bax in rats exposed to induced gastric ulcer and treated with *S. persica* extract. (a) Control group, (b) EtOH-induced gastric ulcer group, (c) EtOH-induced gastric ulcer and treated with *S. persica* at a dose of 200 mg/kg b.w. group, and (d) EtOH-induced gastric ulcer and treated with *S. persica* at a dose of 400 mg/kg b.w. group. Scale bar = 50 *µ*m.

**Figure 6 fig6:**
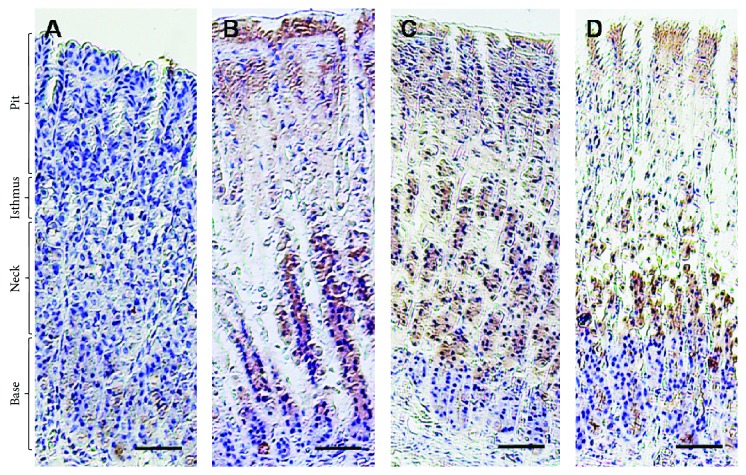
Immunohistochemical reactivity of iNOS in rats exposed to induced gastric ulcer and treated with *S. persica* extract. (a) Control group, (b) EtOH-induced gastric ulcer group, (c) EtOH-induced gastric ulcer and treated with *S. persica* at a dose of 200 mg/kg b.w. group, and (d) EtOH-induced gastric ulcer and treated by *S. persica* at a dose of 400 mg/kg b.w. group. Scale bar = 50 *µ*m.

**Figure 7:**
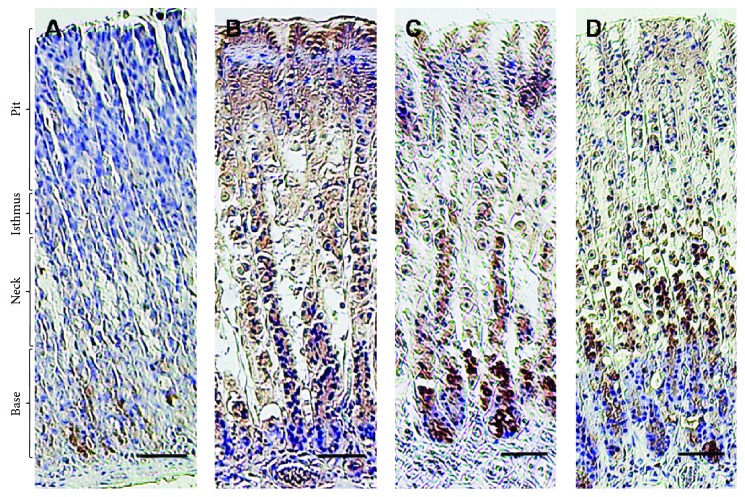
Immunohistochemical reactivity of IL-1*β* in rats exposed to induced gastric ulcer and treated with *S. persica* extract. (a) Control group, (b) EtOH-induced gastric ulcer group, (c) EtOH-induced gastric ulcer and treated with *S. persica* at a dose of 200 mg/kg b.w. group, and (d) EtOH-induced gastric ulcer and treated with *S. persica* at a dose of 400 mg/kg b.w. group. Scale bar = 50 *µ*m.

**Figure 8:**
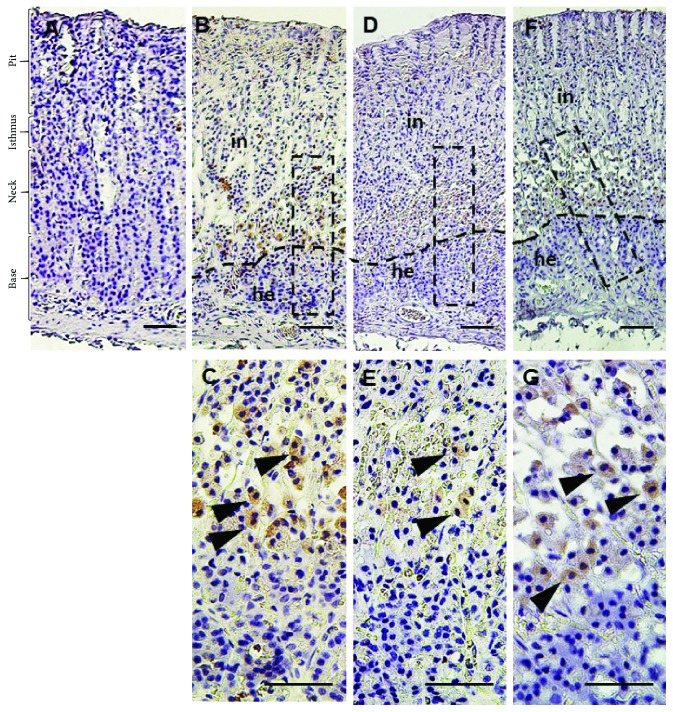
Immunohistochemical reactivity of CD3 in rats exposed to induced gastric ulcer and treated with *S. persica* extract. (a) Control group, (b-c) EtOH-induced gastric ulcer group, (d-e) EtOH-induced gastric ulcer and treated with *S. persica* at a dose of 200 mg/kg b.w. group, and (f-g) EtOH-induced gastric ulcer and treated with *S. persica* at a dose of 400 mg/kg b.w. group. Scale bar = 50 *µ*m.

Immunohistochemical analysis revealed an overexpression of Bax, IL-1*β*, iNOS, and CD3 proteins in the ethanol-treated group as indicated by high distribution of IL-1*β*-, Bax-, and iNOS-immunopositive cells in the base, neck, isthmus, and mucous surface of the pit region as compared to negative or low distribution of immunopositive cells in the control group (Figures 5 [Fig fig6]–7), while CD3-immunopositive cells distributed at the margin of the inflammation that was extensive in the alcohol group penetrated all epithelial layers (Figure 8). Interestingly, pretreatment with SP extract significantly reduced the immunopositive staining of IL-1*β*, Bax, CD3, and eNOS in a dose-dependent manner in the EtOH group, elucidating reduced expression of these proteins.

## 4. Discussion

The current study highlights the antiulcerative effect of SP aqueous extract against EtOH-induced gastric ulceration in rats. This antiulcerative potential might be due to the antioxidant ingredients found in SP aqueous extract such as lycopene [31], *α*-linolenic acid [32], oleic acid [33], lycoxanthin [34], and retinoic acid [35].

Short-term exposure to ethanol-induced gastric damage, hemorrhagic erosions, and increased gastric ulcer index (UI) was confirmed by our histological findings: hemorrhagic degeneration, submucosal edema, shedding of gastric pits, and decreased glycoproteins in the gastric mucosal surface. These results are similar to those obtained by Yang et al. [36] who stated that ethanol had an immense effect on the gastric mucosa represented by severe hyperemia, inflammatory cell infiltration, loss of epithelial cells, and cell erosions. In addition to hemorrhage, epithelial exfoliation, submucosal edema [37], and mucosal friability [38] were recognized. In a recent study, interrupted gastric mucosa, neutrophil infiltration, glandular cell nuclear intensification, and acute hemorrhage were the major histological findings following ethanol exposure in rats [39].

The collective mechanisms implicated in the gastric damaging effect induced by ethanol are due to the perturbation of the antioxidant system, recruitment of inflammatory cascade and apoptosis, and disturbance of nitric oxide synthases. Results of the present study revealed that ethanol stimulated slight production of MDA accompanied by the minimization of both enzymatic and nonenzymatic antioxidants in gastric tissues. Antonisamy et al. [5] revealed that the imbalance between prooxidant and antioxidant molecules is the major contributor of ethanol-induced gastric damage. Park et al. [25] reported that ethanol had dual effects on the gastric mucosa: direct action through damaging the mucosal membranes, cytotoxic dehydration, and generation of inflammatory signaling pathways and an indirect action via neutrophil infiltration with subsequent inflammation and induction of oxidative stress and apoptosis. Alcohol consumption catalyzed the formation of MDA in the gastric tissue with a reduction in SOD, CAT enzymatic activities [40], and GPX activity [41]. Treatment with SP extract caused a decline in the gastric MDA level and enhanced the enzymatic and nonenzymatic antioxidants. The antioxidant activity of SP may be attributed to its high content of furan derivatives, vitamin C, tannins, saponins, and flavonoids [42, 43].

The data of the current study showed an upregulation of gene expression of proinflammatory cytokines: TNF-*α* and IL-1*β* in the gastric mucosa of the EtOH-treated rats. In the same context, Katary and Salahuddin [44] reported the enhancement of gene expression and mucosal levels of TNF-*α* along with increased mucosal levels of IL-1*β* after ethanol consumption in rats. The gastric inflammatory condition has been associated with the release of TNF-*α*, which activates the immune cells and other proinflammatory cytokines and increases the NF-*κ*B expression [45]. TNF-*α* could trigger gastric tissue damage mediated through the activation of neutrophil migration into the gastric tissue associated with retardation of gastric ulcer healing [37]. Inevitably, alcohol resulted in the augmentation of the proinflammatory cytokines: TNF-*α* participating in gastric ulcer via boosting apoptosis, NF-*κ*B, iNOS, and neutrophil infiltration and IL-1*β* arousing the oxidative stress inflicting the gastric damage [46]. Meanwhile, TNF-*α* promotes the release of oxygen free radicals and other proinflammatory cytokines, causing destruction of cell membrane stability and leading to gastric tissue injuries [36].

TGF-*β*1 coordinates different signaling pathways including adhesion, cell proliferation, angiogenesis, and production of extracellular matrix components [47]. Our data revealed low expression of TGF-*β*1 in the EtOH-treated group, while SP extract mitigated the inflammatory effect of ethanol on the gastric tissue, which is in compliance with the results of Monforte et al. [17] who showed that the antiulcer activity of SP decoction against acetylsalicylic acid-induced gastric ulcer in rats resulted in a significant reduction of UI via anti-inflammatory activity. Furthermore, the serum proinflammatory cytokine IL-1*β*, IL-6, and TNF-*α* levels were significantly decreased following the administration of ethyl acetate extract of SP in a gastric ulcer model [20].

Apoptosis induction was evidenced in the current study by increased protein expression of the proapoptotic Bax after EtOH exposure. Ye et al. [48] reported that the gastric damage induced by ethanol might be due, to a certain extent, to the enhancement of the apoptotic pathway. The proapoptotic protein Bax signals the initiation of apoptosis [4], causing cytochrome-C release followed by caspase cascade activation and finally apoptotic cell death [49]. Notably, Al Batran et al. [50] implied that ethanol-triggered gastric injury was in part due to the enhancement of apoptosis in subsequent mucosal epithelial cell loss. The attenuation of lipid peroxidation and TNF-*α* secretion resulted in inhibition of the gastric apoptosis [4]. The antioxidative and anti-inflammatory activities of SP reflected the inhibition of the apoptotic pathway, providing another explanation of its antiulcer activity.

Ethanol evoked the protein expression of iNOS and the decline of the gene expression of eNOS in gastric tissue. A high concentration of NO generated from iNOS was involved in the gut tissue damage during the inflammatory conditions [51]. Nagai et al. [52] indicated that iNOS-produced NO had a critical role in the enhancement of gastric ulcer. Activation of iNOS expression was associated with gastric ulcer and chronic ulcerative colitis in affected patients, suggesting a detrimental effect due to the excessive production of NO on the pathogenesis of these conditions [53]. In contrast, eNOS-derived NO plays a central role in gastric ulcer healing via the maintenance of gastric epithelium, mucosal blood flow, and mucus secretion and synthesis [54]. The present results are comparable to those of Pan et al. [55] who reported that ethanol activated iNOS and the inhibitory effect on eNOS gene expression. Furthermore, ethanol provoked the gene expression of TNF-*α*, IL-1*β*, and iNOS, inflicting the immense effect of the generated NO on gastric ulcer formation [56]. This phenomenon was initiated via the NF-*κ*B pathway where the production of proinflammatory cytokines TNF-*α* and IL-1*β*, upregulation of iNOS gene expression, and release of NO were enhanced by activated NF-*κ*B during gastric ulcer formation [57]. However, pretreatment with SP extract significantly reduced the protein expression of iNOS and upregulated eNOS gene expression in gastric tissue exposed to EtOH, indicating an antiulcerative effect. NO derived from highly expressed iNOS in the ulcerated stomach had no role in the healing process modulation. On the contrary, eNOS-originated NO might enhance the formation of new blood vessels assisting the gastric ulcer healing [11]. This hypothesis suggested the involvement of NO generated from eNOS isoforms in the healing process of gastric ulcers. This mechanism emphasizes the pivotal role that NO plays in the liberation of vasoactive peptides and in the stimulation of cGMP in gastric tissue [58]. NO-stimulated cGMP led to relaxation of mouse gastric smooth muscles and prevented the cytotoxic effect of EtOH on the gastric parietal cells [59]. Also, eNOS-originated NO established the protection and healing of gastric ulcer via augmentation of mucus and bicarbonate secretions, promotion of blood flow, and angiogenesis [60]. CD3 proteins are receptors characteristic for T lymphocyte populations. Bamford et al. [61] detected an increase of CD3 expression in gastric inflammation caused by infection of *H. pylori.* Our results showed the increase of intensity of CD3-expressed cells in the EtOH-treated group that decreased in the SP-treated groups suggesting the effective role of SP in decreasing the symptoms of inflammation that resulted from alcohol exposure. This supports our histological PAS staining that was used to evaluate the level of glycoprotein, mucin, and subsequent mucus content; intense mucin staining in the mucosal cell layer reflects high mucus content in the SP-treated groups, suggesting a protective effect.

## 5. Conclusion

SP aqueous extract alleviated serious gastric mucosal ulcerations induced by ethanol and emphasized its efficacy as an antiulcer protectant. The underlying mechanism of its activity is through the enhancement of the antioxidative defense system, minimization of proinflammatory cytokines and apoptotic pathway, augmentation of mucus content, and redesign of the NOS isoforms supporting the antioxidant, anti-inflammatory, and antiapoptotic effects of SP favoring the healing and prevention of gastric ulcers.

## Figures and Tables

**Table 1:** Ingredients of basal diet.

Ingredients	g/kg diet
Corn flour	529.5
Casein	200
Sucrose	100
Soybean oil	70
Cellulose	50
Mineral mix	35
Vitamin mix	10
L-Cysteine	3
Choline	2.5

**Table 2:** Primer sequences.

Gene symbol	Gene description	GenBank accession number	Sequence	Annealing temperature (°C)
Actb	*β*-Actin	NM_031144.3	F: TGTTGTCCCTGTATGCCTCT R: TAATGTCACGCACGATTTCC	60
eNOS	Endothelial nitric oxide synthase	NC_005103.4	F: TCTTCAAGGACCTACCTCAGGC R: GCTAAGGCAAAGCTGCTAGGTC	60
TGF-*β*1	Transforming growth factor-*β*1	NM_021578.2	F: CCAACTACTGCTTCAGCTCCACA R: TGTACTGTGTGTCCAGGCTCCAAA	58
TNF-*α*	Tumor necrosis factor-*α*	NM_012675.3	F: GACCCTCACACTCAGATCATCTTCT R: TTGTCTTTGAGATCCATGCCATT	60
IL-1*β*	Interleukin-1*β*	NM_031512.2	F: CACCTCTCAAGCAGAGCACAG R: GGGTTCCATGGTGAAGTCAAC	60

**Table 3:** Phytochemical analysis of *S. persica* extract by GC-MS.

Retention time (min)	Phytochemicals	Area (%)
5.52	Chavicol	1.18
10.18	Oleic acid	1.97
12.31	3-Penten-2-one	4.24
15.58	Tristrimethylsilyl ether derivative of 1,25-dihydroxyvitamin D2	3.94
16.24	Retinoic acid	1.31
16.78	Palmitic acid	13.19
17.27	Androst-7-ene-6,17-dione	9.56
19.05	Methyl alpha-D-glucopyranoside	4.09
19.32	*α*-Linolenic acid	1.77
20.83	Tributyl acetylcitrate	5.24
23.78	Hexa-t-butylselenatrisiletane	6.97
26.33	Lycopene	16.56
27.45	Pregn-16-ene-11,14,18,20-tetrol	1.73
28.46	Lycoxanthin	1.61
	Ingredients less than 1.00%	26.64

**Table 4:** Oxidative stress and antioxidative profile in ethanol-induced gastric ulcer rat model pretreated with *S. persica* extract.

	MDA (nmol/g)	GSH (*μ*mol/g)	T.SOD (U/g)	CAT (U/g)	GPX (U/g)	GST (U/g)
Control	101.3 ± 8.2^a^	16.8 ± 1.6^c^	75.4 ± 7.8^c^	11.2 ± 2.6^c^	46.9 ± 7.6^c^	143.2 ± 12.4^c^
EtOH	111.5 ± 7.6^a^	14.2 ± 1.4^d^	70.5 ± 5.4^c^	9.8 ± 1.1^d^	41.7 ± 8.9^c^	131.8 ± 15.8^c^
EtOH + SP200	83.7 ± 8.9^b^	18.6 ± 2.1^b^	85.4 ± 7.1^b^	13.6 ± 2.3^b^	63.8 ± 8.7^b^	165.7 ± 13.3^b^
EtOH + SP400	74.3 ± 9.1^b^	22.9 ± 1.9^a^	96.8 ± 6.4^a^	16.7 ± 2.2^a^	72.9 ± 6.3^a^	181.2 ± 11.8^a^

Values are expressed as mean ± SEM. The means within the same column carrying different superscript letters are significantly different at *P* < 0.05 as determined with one-way ANOVA, followed by Duncan's post hoc test multiple comparisons. EtOH: ethanol-induced gastric ulcer group; EtOH + SP200: ethanol-induced gastric ulcer pretreated with *S. persica* extract at a dose of 200 mg/kg b.w.; EtOH + SP400: ethanol-induced gastric ulcer pretreated with *S. persica* extract at a dose of 400 mg/kg b.w.

## Data Availability

All data generated or analyzed during this study are included in this article and its supplementary information files.

## Supplementary Materials

Supplementary Materials Figure S1: GC-MS chromatogram showing the area % of *S. persica* extract phytoconstituents at different retention times.
