# Supplementary figures and images for: Protective Effects of Miswak (Salvadora persica) against Experimentally Induced Gastric Ulcers in Rats
Source: Oxid Med Cell Longev. 2018 Jul 9;2018:6703296. doi: 10.1155/2018/6703296 (PMC6079327; doi:10.1155/2018/6703296)

## Supplemental Figure Legend

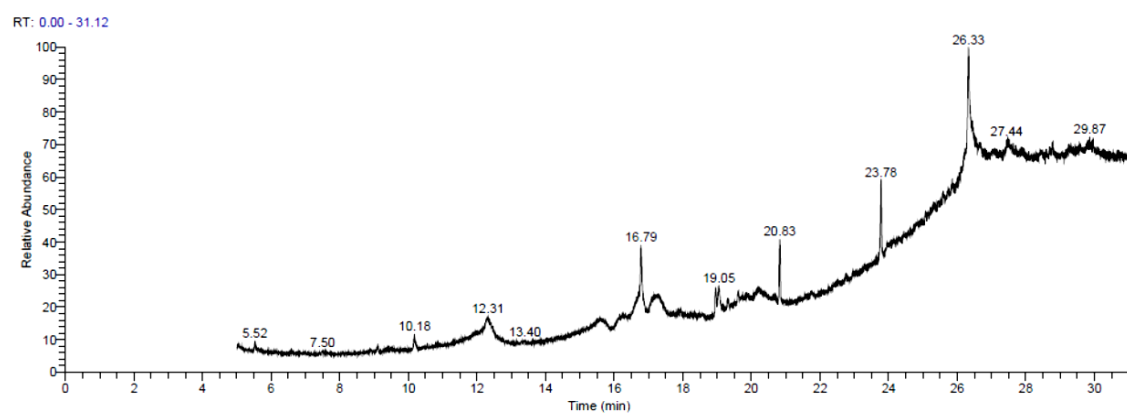

S1. Fig.1. Chromatogram of GC-MS analysis of *S. persica* extract

Supplement: Supplementary Materials — Figure S1: GC-MS chromatogram showing the area % of S. persica extract phytoconstituents at different retention times. [file 6703296.f1.pdf]
